# Clitoral Epidermal Inclusion Cyst in a 12-Year-Old Girl

**DOI:** 10.7759/cureus.91129

**Published:** 2025-08-27

**Authors:** Shoag Albugami, Meral F Alzimam, Abdulaziz K Aldosari, Mohanned S Aljohany, Mohammad Alonazi

**Affiliations:** 1 General Surgery, Prince Sultan Military Medical City, Riyadh, SAU; 2 Pediatric Surgery, King Faisal Specialist Hospital and Research Centre, Riyadh, SAU; 3 Pediatric Surgery, Prince Sultan Military Medical City, Riyadh, SAU

**Keywords:** clitoral epidermoid inclusion cyst, epidermal clitoral inclusion cyst, female genital mutilation, fgm, inclusion cyst

## Abstract

Female genital mutilation (FGM) is a procedure that involves partial or total removal of the external female genitalia; usually, it is done by a non-professional practitioner. Here, we present a case of a 12-year-old girl with type 1 diabetes who was found to have a clitoral cyst several years after undergoing FGM (local/traditional circumcision). Unlike many cases that remain unnoticed until adolescence or adulthood, this patient came to medical attention earlier. She underwent elective cyst excision, with preservation of as much of the clitoral remnant as possible. The clitoral cyst was an epidermal inclusion cyst on histopathological examination. The patient was seen in the clinic three weeks after the procedure. She was doing fine with no complaint. Her examination showed a healed, dry, and clean wound, and she was discharged from the clinic.

## Introduction

Female genital mutilation (FGM) is defined by the World Health Organization (WHO) as procedures that involve partial or total removal of the external female genitalia or other injury to the female genital organs for non-medical reasons [1]. Although FGM is illegal in many countries, it is still practiced by non-medical professionals in Africa and the Middle East. In Saudi Arabia, few studies reported that FGM is practiced in Jeddah and the Hail region by Saudi and non-Saudi residents [2].

A study published in 2019 investigated the prevalence of FGM among residents under 18 years of age in Hail. The findings revealed that approximately 80.3% had undergone circumcision, with 90.4% of the procedures performed by medical professionals. The study also indicated that FGM was more commonly practiced in families with lower educational levels and socio-economic status. Despite this study, there remains a significant lack of comprehensive research on the prevalence and complications of FGM in Saudi Arabia, highlighting the need for further investigation in this area [2].

The WHO classified FGM into four types [1,3]. Type 1 involves the partial or total removal of the clitoral glans (the external and visible part of the clitoris, which is a sensitive part of the female genitals) and/or the prepuce/clitoral hood (the fold of skin surrounding the clitoral glans). Type 2 is the partial or total removal of the clitoral glans and the labia minora (the inner folds of the vulva), with or without the removal of the labia majora (the outer folds of the skin of the vulva). Type 3 is also known as infibulation, which is the narrowing of the vaginal opening through the creation of a covering seal. The seal is formed by cutting and repositioning the labia minora or labia majora, sometimes through stitching, with or without removal of the clitoral prepuce/clitoral hood and glans. Type 4 includes all other harmful procedures to the female genitalia for non-medical purposes, e.g., pricking, piercing, incising, scraping, and cauterizing the genital area [1].

FGM can lead to complications that appear both early in childhood and later in adulthood. Early complications may include bleeding and infections, while long-term effects can involve urinary and menstrual difficulties, the development of epidermal cysts, and complications during childbirth [1,4,5]. Here, we present a case of a 12-year-old girl who underwent type 1 FGM as a neonate and later came to our clinic with clitoral swelling.

## Case presentation

**Figure 1:**
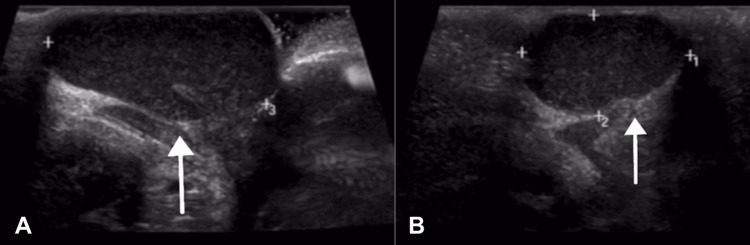
Ultrasonography of the clitoral swelling showing a hypoechoic lesion measuring 2.6 x 1.6 x 3.5 cm (A, B).

**Figure 2:**
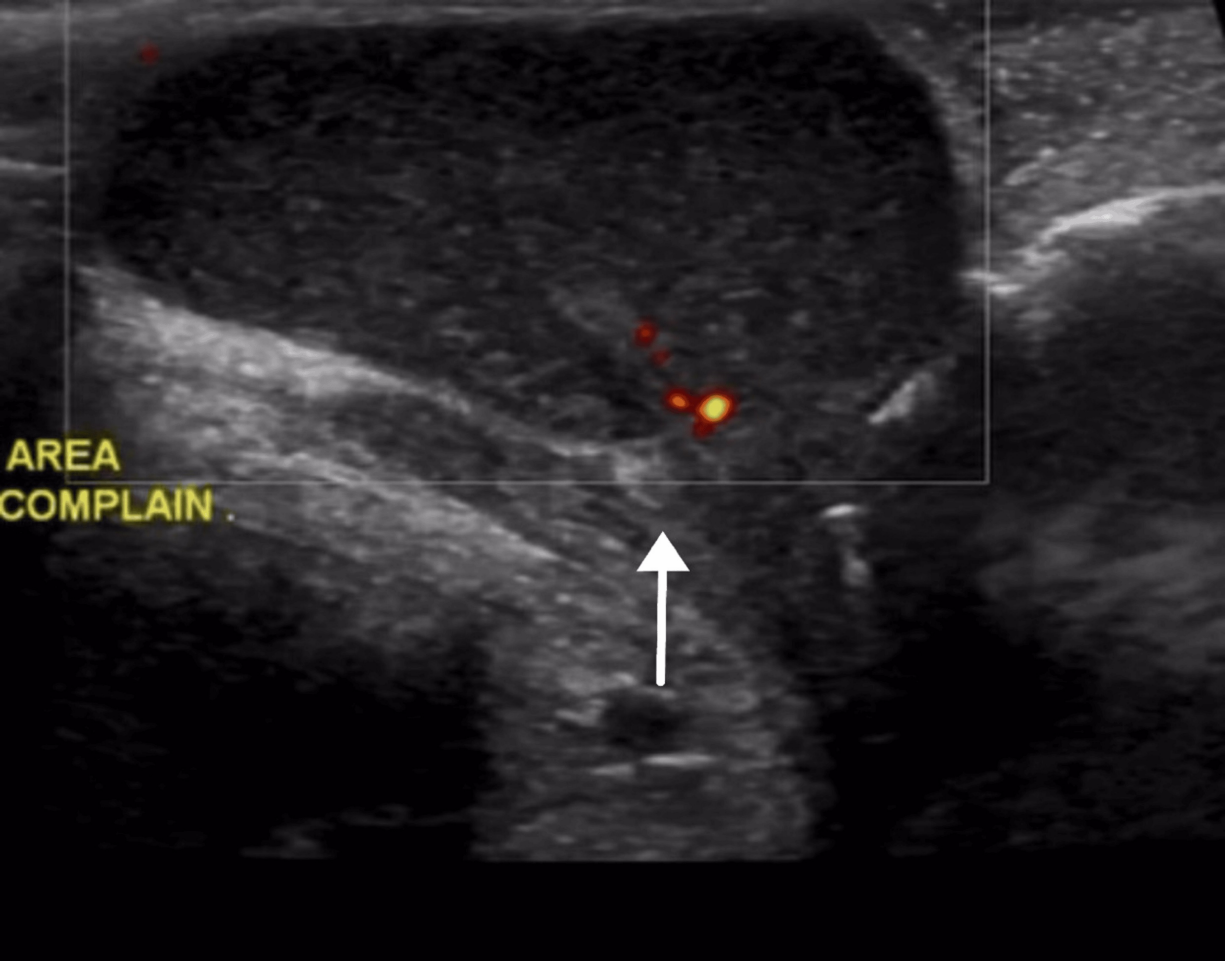
Color Doppler shows no Doppler signal.

This case involves a 12-year-old girl who presented to our outpatient clinic with clitoral swelling for five years. She had a circumcision done in the neonatal period by a local non-professional practitioner. The swelling had been increasing in size over time, and there was no history of skin changes, pain, or discharge associated with it. She is a known case of type I diabetes mellitus with no significant past surgical history. On examination, there was a soft, nontender, partially mobile swelling at the site of the clitoris measuring 2 × 3 cm, clearly separated from the vestibule and urethra. Ultrasonography of the swelling showed a hypoechoic focal lesion measuring 2.6 × 1.6 × 3.5 cm, corresponding to a volume of 7.4 mL with internal echoes, posterior acoustic enhancement, and no definite sign of internal vascularity suggestive of a clitoral cyst (Figures 1, 2).

**Figure 3:**
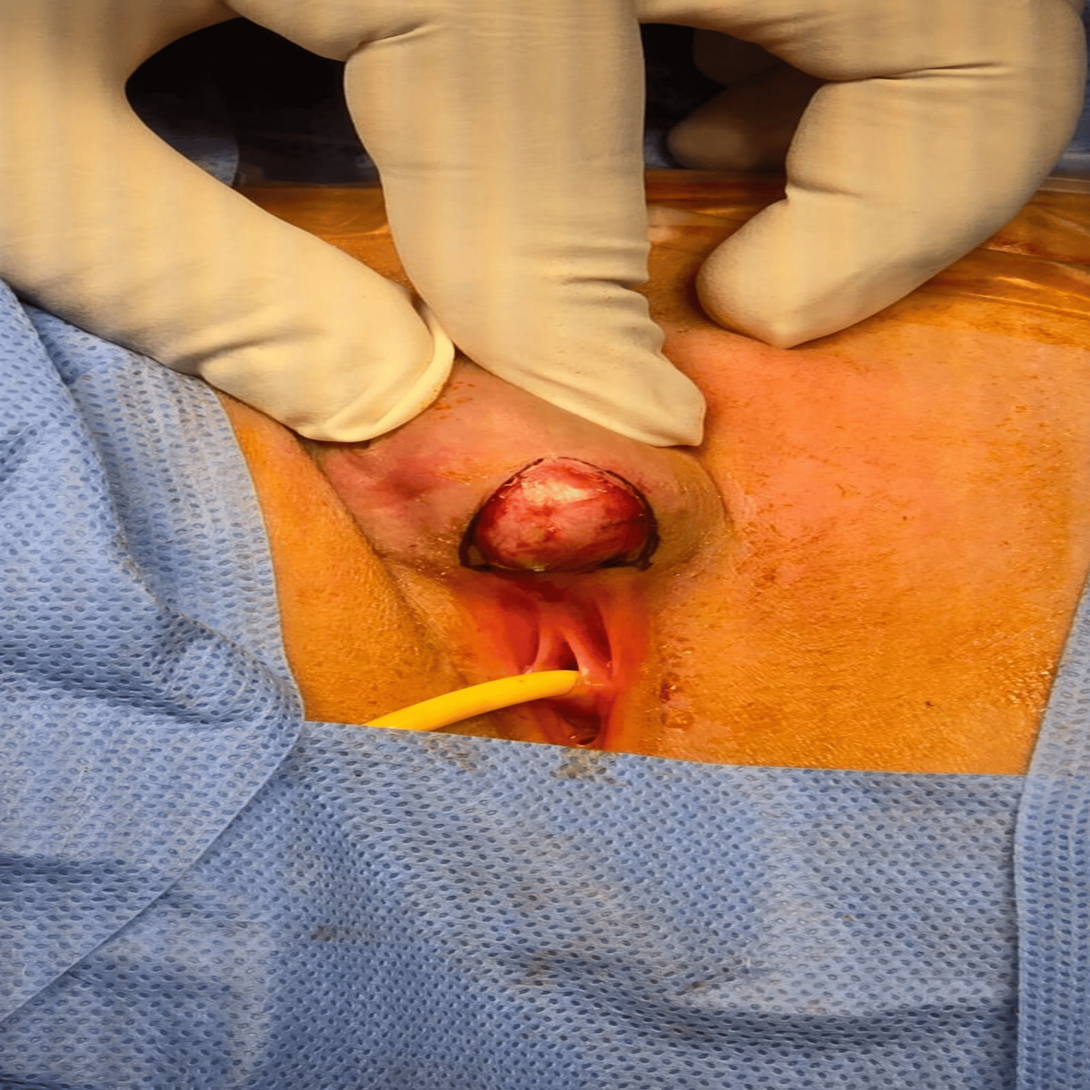
The intraoperative image shows a longitudinal incision at the lower border of the cyst, which extended inferiorly to the upper border of the vestibule.

**Figure 4:**
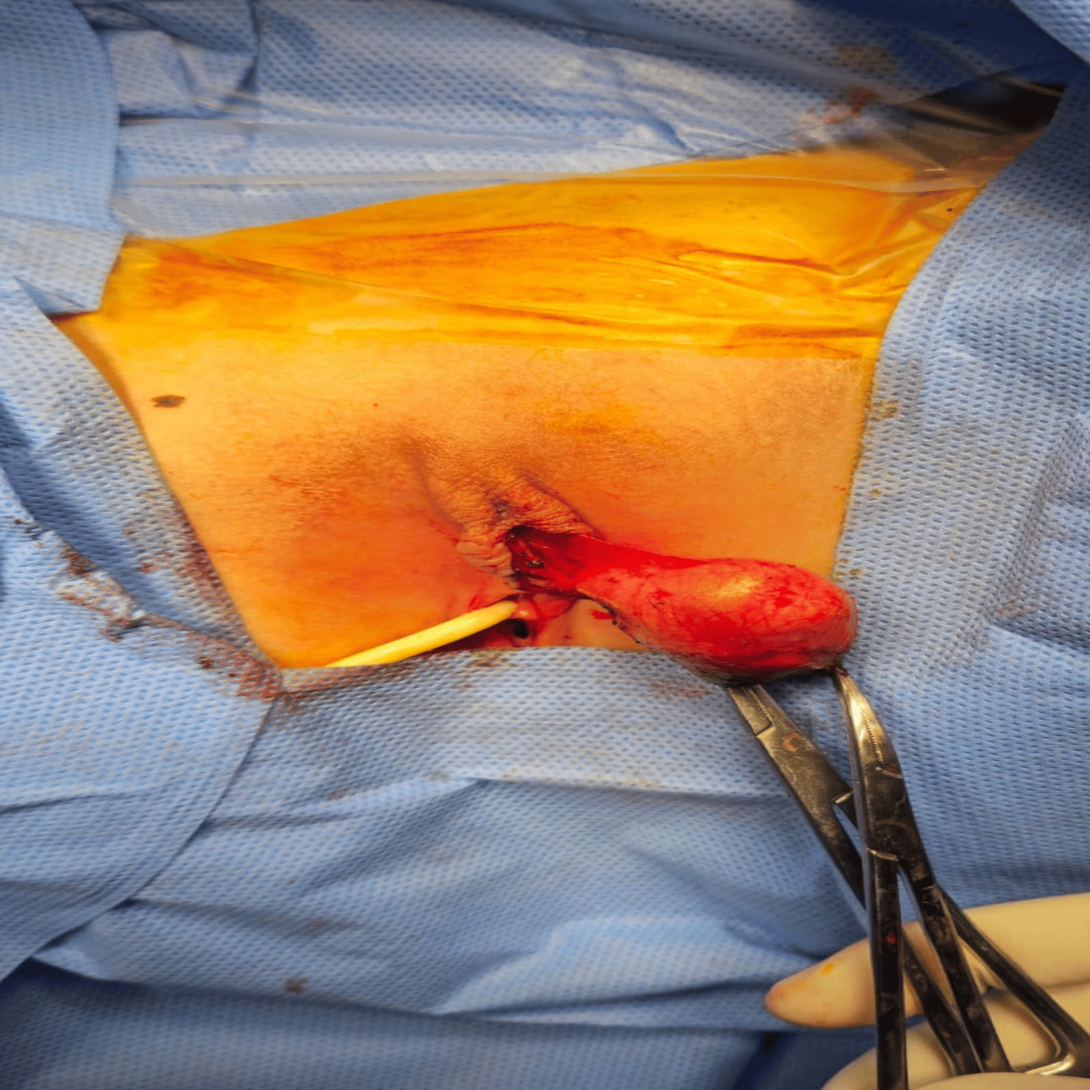
The intraoperative image shows a clitoral cyst dissected free from the remnant of the clitoris.

**Figure 5:**
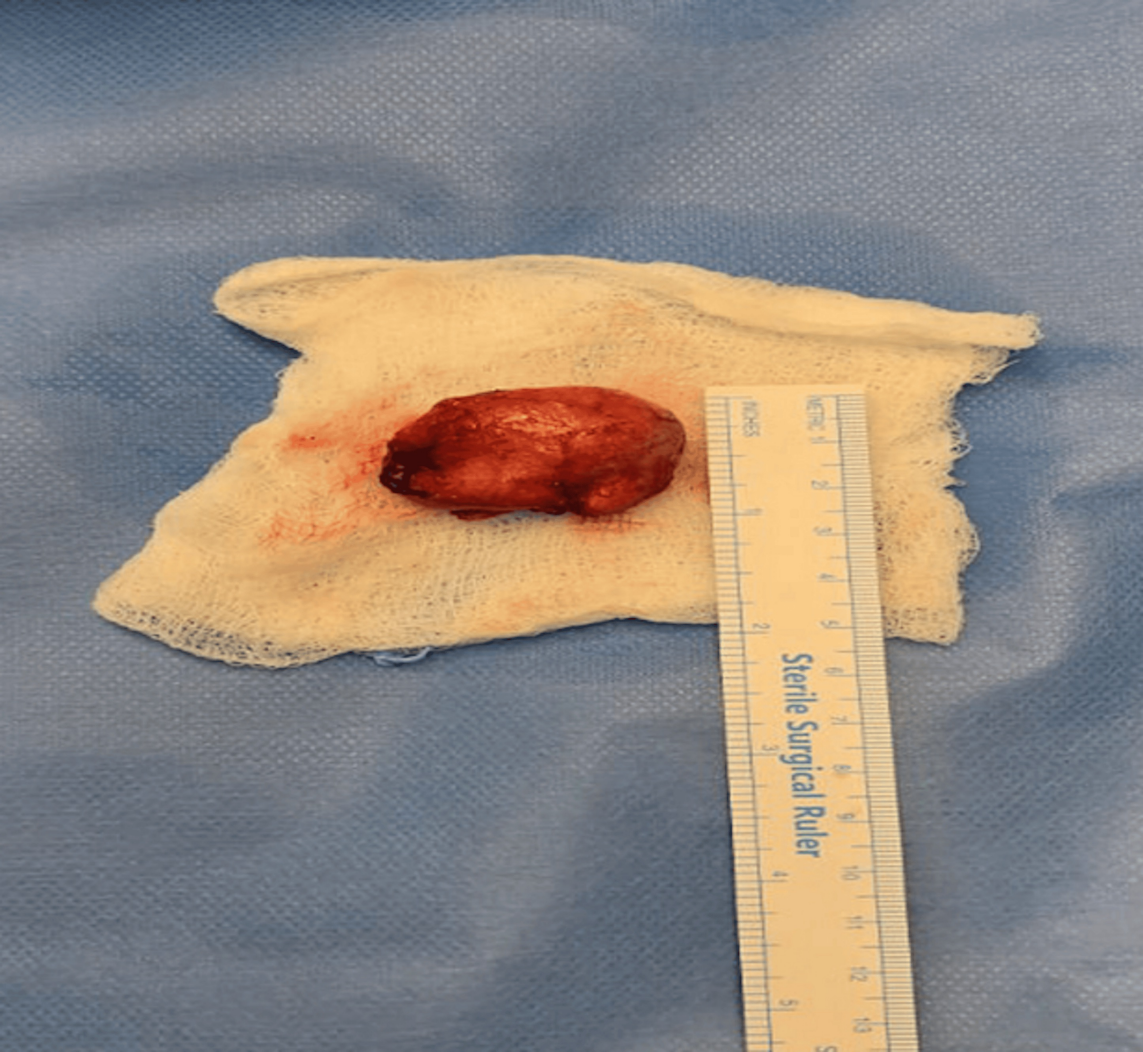
The intraoperative image shows an excised clitoral cyst.

**Figure 6:**
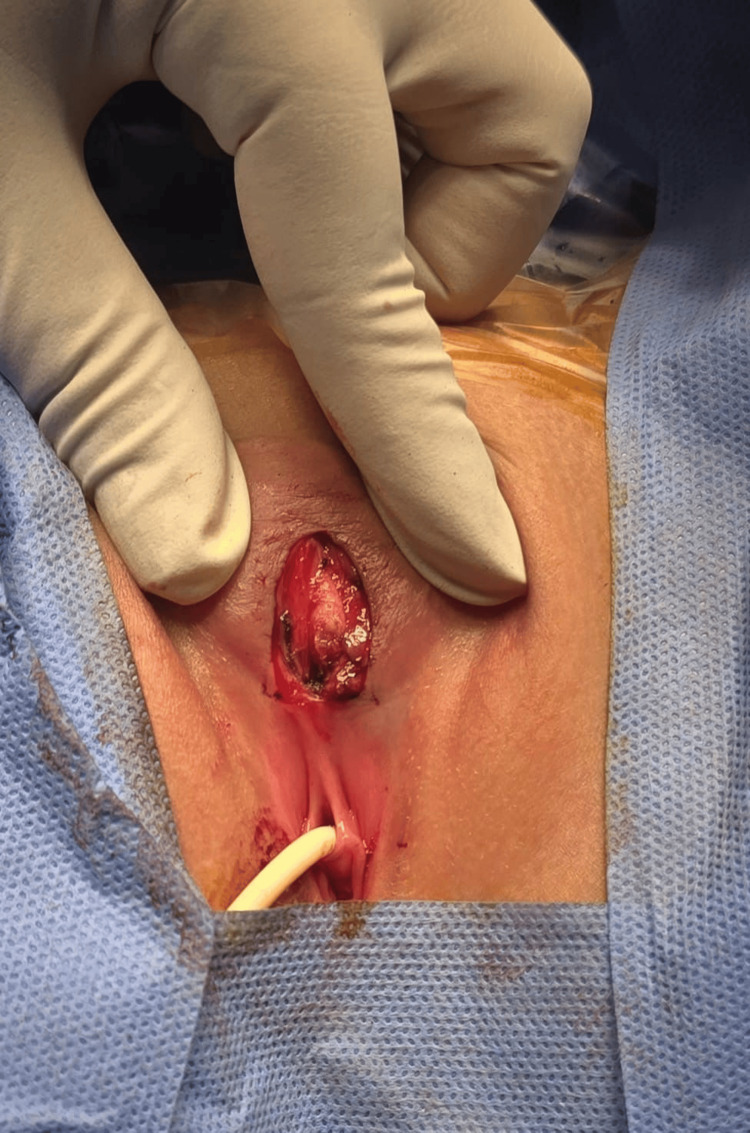
The intraoperative image shows a remnant of the clitoris post-complete excision of the cyst.

Accordingly, the patient was admitted electively for surgical excision (Figures 3-6).

The patient was kept for one day postoperatively and discharged home in good condition. The histopathological examination revealed that the clitoral cyst was an epidermal inclusion cyst. The patient was seen in the clinic three weeks after the procedure. She was doing fine with no complaint; her examination showed a healed, dry, and clean wound, and she was discharged from the clinic.

## Discussion

Clitoral epidermoid inclusion cysts are an uncommon but important complication seen after FGM, particularly type I, where part or all of the clitoris is removed. These cysts tend to develop slowly over time as a result of skin cells being trapped beneath the surface during the trauma, gradually forming a lump filled with keratin and sebaceous material [1-7].

In our case, a 12-year-old girl with type 1 diabetes was found to have a clitoral cyst several years after undergoing FGM (local/traditional circumcision). Unlike many cases that remain unnoticed until adolescence or adulthood, this patient came to medical attention earlier. This was likely due to the added medical attention related to her diabetes and proactive concern from her caregivers. Hormonal changes during puberty often accelerate the growth of these cysts, and estrogen in particular is thought to play a role in stimulating the trapped skin cells [6-8]. Osifo and colleagues reported that many girls do not seek help until their late teens, when the cysts become uncomfortable or visibly concerning [8].

In contrast, some patients may go years without a diagnosis. Rizk et al. and Rouzi et al. described adults who lived with these cysts for decades before presenting for evaluation, often due to embarrassment, fear of stigma, or lack of access to care [5,6]. DiCarlo-Meacham et al. described a striking case where the cyst not only distorted anatomy but also led to sexual dysfunction in the form of anorgasmia, highlighting how critical early recognition and treatment can be [9].

When evaluating clitoral masses in young patients, it is important to consider a wide range of possibilities, such as hormonal disorders, congenital differences, trauma, or tumors. However, a history of FGM combined with a painless, slow-growing lump should raise a strong suspicion for an epidermoid inclusion cyst [6,10]. Imaging is not always necessary, but when used, MRI can help define the anatomy and support safer surgery. Johnson et al. demonstrated that using a small MR coil can help visualize important structures like the nerves and blood vessels of the clitoris, which are vital for preserving future sexual function [7].

Surgical removal is usually curative, and when performed carefully, it allows for the preservation of remaining clitoral tissue and sensation. In our case, early diagnosis and timely surgery allowed for a straightforward excision. This outcome contrasts with cases, such as those described by Osifo, where the cysts had grown so large that they involved nearby structures and required more extensive operations [8].

As DiCarlo-Meacham et al. emphasized, these cysts can have a profound effect on sexual health and emotional well-being, even in patients who are not yet sexually active. Their review suggests that early treatment may help avoid the psychological and physical burdens seen in older patients [9].

This case serves as an important reminder of the value of routine genital exams in pediatric care, particularly in patients with known or suspected FGM. While efforts to eliminate FGM continue, healthcare providers must be prepared to recognize and compassionately manage the consequences of this practice, ensuring that girls receive timely, respectful, and appropriate care.

## Conclusions

Clitoral epidermoid inclusion cysts are a complication seen after FGM. These cysts result from skin cells being trapped beneath the surface during trauma, gradually forming a lump filled with keratin and sebaceous material. This case serves as an important reminder of the value of routine genital exams in pediatric care, particularly in patients with known or suspected FGM.
